# Planetary health: The need for a paradigm shift

**DOI:** 10.1093/biosci/biad107

**Published:** 2023-11-29

**Authors:** Pim Martens

**Affiliations:** System Earth Science Institute; The University College Venlo, part of Maastricht University, Maastricht, The Netherlands

It is common knowledge that the Earth has changed considerably in recent centuries because of increasing economic and population growth. These changes have been so significant in recent years that scientists see the beginning of a new geological era, the Anthropocene, an era in which man has become the most important factor in degrading the Earth (Steffen et al. 2007). These changes affect not only our atmosphere but also our soil, water, and biodiversity.

Our food but also clean water and clean air depend on nature. Our energy sources, raw materials, building materials, and medicines come from nature too. We may have a cure for cancer within reach if we make an effort to protect our ecosystems and plant species. Nature and biodiversity ensure our health, well-being, and quality of life. For example, people feel more comfortable in nature. Various lifestyles and cultural practices could not survive without the nature on which they depend. Maintaining biodiversity is important for our health and for the health of the planet.

## Planetary health

One of the concepts that acts as an umbrella describing changes on our planet in relation to our health is planetary health (Whitmee et al. 2015). However, the concept as launched a few years ago is too human oriented (Martens 2023). The emphasis is mainly on the consequences for our health through disturbances in the environment. Later definitions are already better, and the focus is more on the health of our planet, with the realization that human health ultimately depends on the health of the planet.

However the field of planetary health is more than that. It is not only the realization that everything is connected but also the realization that it is not nearly enough to keep the planet as it is. Positive, regenerative development must take place to keep the planet and everything on it healthy. This also includes a different way of dealing with our Earth: a change of perspective. Planetary health can therefore be defined as a holistic systems view of the world, recognizing the interconnectedness of the health and well-being of all life on Earth and the need to regenerate natural systems, rather than simply minimizing harm.

In recent years, the term *planetary health* has gained popularity within the medical and health sciences. And although the term *planetary health* is relatively new, some underlying concepts are not. An important example is *global health*, in which the population's health is studied globally, or *one health*, which recognizes the interconnectedness among humans, animals, plants, and their environment.

Another way to look at planetary health is from an ecological perspective. For example, ecohealth looks at how changes in the Earth's ecosystems affect human health. Another concept, planetary boundaries, was introduced in 2009 (Rockström et al. 2009). Nine planetary boundaries were established within which humanity must remain without causing further damage to our planet.

However, planetary health does not replace these closely related concepts but, instead, complements them. Even more so than the other disciplines just mentioned, the field of planetary health will be interdisciplinary and transdisciplinary. Given the complexity of the problems we face, it is imperative that doctors, ecologists, social scientists, agricultural scientists, and the rest of us work together.

There must also be cooperation outside the academic walls with governments, companies, and other organizations—so-called transdisciplinary research. But to ensure the health of our Earth, planetary health will also need to embrace a holistic and perhaps even a spiritual approach and integrate it with scientific disciplines. One example of nonacademic knowledge that can play an essential role is the knowledge inherited by indigenous communities.

## Indigenous knowledge

Globally, indigenous peoples and local communities play an important role in the management, conservation, and sustainable use of biodiversity and nature. Many indigenous communities live in areas of high biodiversity, where living in harmony with nature is essential for survival. These communities have strong ties to their territory and apply indigenous knowledge to protect, manage, and use the natural resources in these areas. It has been shown that the ecosystems and species in areas managed by indigenous peoples are often less threatened than in other areas (Sze et al. 2022). And this is just one of the examples where we can learn from indigenous knowledge.

The two-eyed seeing principle calls for the art of bundling all available knowledge and skills. This can apply everywhere in the world (“We are all indigenous to Mother Earth”; Phil Lane Jr., personal communication, 31 August 2020), by integrating the knowledge of, among others, farmers and civilians—in other words, the local indigenous people. We call this transdisciplinary research. If we also keep in mind the aforementioned definition of planetary health and start to see it as two eyed, our cities and countryside may be able to become planetarily healthy in the future.

Hand in hand with transdisciplinary research goes repairing the damage that has been done. We can do this in different ways, but I would like to explain one way in more detail. Nature-based solutions harness nature and the power of healthy ecosystems to protect people, produce food, and secure a stable and biodiverse future.

So transdisciplinary scientific research, using indigenous knowledge that is present everywhere on Earth, as well as restoring nature, is needed. The final element central to planetary health is change.

## Paradigm shift

In these times when influencers, movie stars, and the media increasingly determine how scientific knowledge should be interpreted, we, as scientists, also have to assume a different role. Despite the many pitfalls and the resistance of climate sceptics and people suffering from the shifting-baseline syndrome (Pauly 1995), we, as scientists, can no longer hide behind our academic walls. As scientivists—*scientivists* are scientist–activists, people that are engaged in a systematic activity to acquire knowledge (the science part) to promote, impede, or direct societal change (the activist part)—we must take a more active social role to bring about the necessary change.

Change is also needed in the education we provide. At all levels, most students and teachers are amateurs regarding the planetary health crisis. There is a great need for students who can regenerate our Earth and reshape it in every way. For example, education that still embraces classical economic theories has long ceased to suffice. The current economy, with a strong belief in the efficiency of the private sector and the market mechanism, is one of the causes of today's global environmental problems. But most education on circularity also has serious shortcomings. In the case of circularity, the solutions are mainly sought in technology, in particular, and not from, for example, the social or cultural angle.

So we also need to train students with regenerative education. To restore the health of our planet, we need more systems thinkers and doers. The next generation of students will have to become the regeneration, a generation that combines theory and practice with a long-term vision and integrates different disciplines.

## Conclusions

Although some people believe the Earth would be better off without humans, I don't think so. Of course, certain systems we have created are not healthy—such as intensive agriculture—but we are not these systems. We can change. The Earth needs us humans. To this end, we should not separate ourselves further from nature—for example, by putting fences around it—but we should learn to live with nature again.

So it has never been more important to fundamentally rethink our relationship with our living planet. However, this cannot be done without thinking differently about our place on Earth. Healthy human societies cannot exist on a dying planet. So we must wonder and reconnect with nature. When you respect the Earth, you respect life and yourself. And that, to me, is the essence of planetary health.

## Author Biography

Pim Martens (p.martens@maastichtuniversity.nl) is affiliated with the System Earth Science Institute and the University College Venlo, part of Maastricht University, in Maastricht, The Netherlands
